# Process Parameter Optimization for Laser Powder Bed Fusion of Fe-Si Alloy Considering Surface Morphology and Track Width of Single Scan Track

**DOI:** 10.3390/ma16247626

**Published:** 2023-12-13

**Authors:** Ho Sung Jang, Su Heon Kim, Geon-Woo Park, Jong Bae Jeon, Donghwi Kim, Dohyung Kim, Wang Ryeol Kim, Yoon Suk Choi, Sunmi Shin

**Affiliations:** 1 Advanced Forming Process R&D Group, Korea Institute of Industrial Technology, Ulsan 44776, Republic of Korea; ggi0505@kitech.re.kr (H.S.J.);; 2 School of Materials Science and Engineering, Pusan National University, Busan 46241, Republic of Korea; 3 Dongnam Regional Division, Korea Institute of Industrial Technology, Yangsan 50623, Republic of Korea; 4 Department of Materials Science and Engineering, Ulsan National Institute of Science and Technology, Ulsan 44919, Republic of Korea; 5 Department of Materials Science and Engineering, Dong-A University, Busan 49315, Republic of Korea; 6 Computational Materials Research Team, Hyundai Motor Group, Uiwang 16082, Republic of Korea

**Keywords:** selective laser melting, silicon steel, additive manufacturing, process optimization, single-track scanning

## Abstract

A laser power bed fusion (L-PBF) manufacturing process was optimized by analyzing the surface morphology and track width *w* of single scan tracks (SSTs) on Fe-3.4wt.%Si. An SST was evaluated under process conditions of laser power *P*, scan speed *V*, and energy density *E* = *P*/*V*. The SST surface shape was mainly affected by *E*; desirable thin and regular tracks were obtained at *E* = 0.3 and 0.4 J/mm. An L-PBF process window was proposed considering the optimal *w* of SST, and the appropriate range of *E* for the alloy was identified to be 0.24 J/mm to 0.49 J/mm. *w* showed a strong relationship with *E* and *V*, and an analytic model was suggested. To verify the process window derived from the appropriate *w* of SST, cubic samples were manufactured with the estimated optimal process conditions. Most samples produced had a high density with a porosity of <1%, and the process window derived from SST *w* data had high reliability. This study presents a comprehensive approach to enhancing additive manufacturing for Fe-3.4Si alloy, offering valuable insights for achieving high-quality samples without the need for time-intensive procedures.

## 1. Introduction

Laser Powder Bed Fusion (L-PBF) is an additive manufacturing process in which a laser is beamed at a powder layer to melt it as desired. L-PBF applied layer-by-layer can produce products with complex shapes, and has potential to be applied to structural metals [1,2] For L-PBF, a layer of powder is spread on the building platform and then irradiated using a laser beam in a single scan track (SST) to melt the powder in a pre-modeled area [3]. After the melted powder solidifies, a new layer of powder is coated on the building platform and the process is repeated until the desired 3D shape is produced.

Selecting appropriate process parameters is crucial for forming regular SSTs and manufacturing high-quality products. Optimizing L-PBF requires understanding the laser absorption, heat transfer, fluid flow, and solidification in the molten pool during single-track laser scanning. The melt pool’s formation results from these complex interactions, making it essential to understand it. Unstable melting can cause discontinuous tracks and irregular widths due to the balling effect [4]. This effect can lead to porosity defects and delamination in the final product. If the material is heated beyond its boiling temperature, the molten pool’s width and depth can increase rapidly due to recoil pressure from evaporation and the Marangoni effect from surface tension [5]. These rapid changes can cause depression and spatter. The correlations among these phenomena depend on the powder properties and process parameters.

The main L-PBF process parameters that affect SST formation are laser power *P* (W), scan speed *V* (mm/s), and their combination, linear energy density *E* = *P*/*V* (J/mm) [6,7,8]. Excessive *E* causes the formation of satellites due to burning and sputtering, which prevents the formation of high-density products. Excessive *E* also forms a deep molten pool and increases crack generation during solidification [9]. However, insufficient *E* can cause the balling phenomenon, and powder particles may not fuse, so the desired physical properties of the products may not be obtained [9,10]. Layer thickness *t* (μm) affects surface accuracy and processing speed; as *t* decreases, the stability of the molten pool increases, and the balling effect decreases [6].

The final product’s quality in L-PBF strongly depends on the interaction between SST pairs in the layers. Hatch spacing (*h* (μm)) is the distance between adjacent SSTs within a layer, determined by the sizes of adjacent melt pools and their overlap degree [11,12]. Decreasing the melt pool size while keeping h constant reduces the remelting range and overlap area. As the overlap area ratio decreases, the coupling between adjacent tracks also diminishes [13]. Low coupling can cause porosity defects, but as the overlap ratio increases, so does the evaporation of molten material. An optimal 30% overlap of the SST width has been reported for L-PBF using stainless steel 316L [6].

Fe-3Si is known for its minimal solid-state deformation during the rapid heating–cooling cycles of L-PBF, making it suitable for PBF applications. Additively manufactured rotors with this alloy showed reduced power loss and improved energy efficiency compared to bulk specimens [14,15]. However, research indicates that the optimal process conditions for L-PBF are narrow, and achieving high-density products is challenging, limiting commercialization. Process optimization studies for the Fe-Si alloy involved producing bulk specimens under various conditions and analyzing defects [16]. Existing SST analysis for process optimization has relied on cross-sectional melt pool analysis and statistical or finite element analysis [17,18]. However, these methods are complex and time-consuming. Therefore, rapid optimization methods and appropriate processing maps are needed. Understanding SST conditions is crucial for these goals.

This paper presents the optimization of an additive manufacturing process by analyzing the track width *w* and morphology of an SST generated by laser on Fe-3.4Si substrate. The focus was on the SST surface shape characteristics and *w* according to the process conditions, rather than on the time-consuming cross-sectional analysis of the shape of the molten pool. This study derived an optimal combination of *P* and *V*. The optimal *w* was predicted under stable SST formation conditions, and the influence of *w* according to each process condition was evaluated. Cubic samples were produced for the analysis of porosity under the derived optimal process conditions.

## 2. Materials and Methods

A silicon steel powder containing 3.4 wt.% Si was produced by gas atomization (Table 1). Particles were spherical with D_10_ = 16.8 μm, D_50_ = 28.7 μm, and D_90_ = 48.6 μm. The SST sample was fabricated using a 3D metal printer (OPM 250L, Sodick Co., Ltd., Kanagawa, Japan) equipped with an ytterbium fiber laser with a wavelength of 1070 nm and maximum power of 500 W. The laser spot has a Gaussian beam cross-section. The L-PBF process was carried out under 1% oxygen in an N_2_ atmosphere. The platform on which the specimens are stacked was maintained at 100 °C to prevent thermal expansion due to the temperature gradient that forms as the temperature of the specimens increases.

The SST was laser-irradiated in a section of the length of 10 mm separated by gaps of 1 mm. The main process conditions considered in this study were 125 ≤ *P* ≤ 200 W, 100 ≤ *V* ≤ 1000 mm/s, and 0.2 ≤ *E* ≤ 0.8 J/mm. *Bs* = 100 μm, *h* = 80 μm, and *t* = 40 μm were held constant (Table 2). These process conditions were set in a wide range by referring to previous studies [16,19,20]. Specifically, *P* and *V* were set variously to clarify the effects of the linear energy input (*E* = *P*/*V*) on SST morphology and *w*.

**Figure 1:**
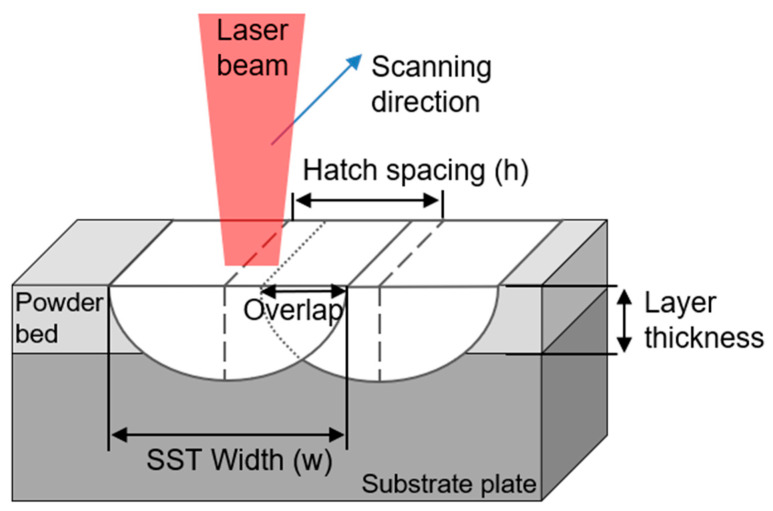
Schematic illustration of horizontal overlap ratio of melt.

The value of *w* of the SST at each process condition was measured, and the morphological characteristics were observed using a digital optical microscope (RH-2000,HIROX Co., Ltd., Tokyo, Japan). Previous studies [6,12] determined that the optimal melt pool overlap is 25 to 35% (Figure 1). It can be calculated as

(1) $$Overlap\;rate(\%)=\frac{h}{w+h}\times 100\%\left(h=\frac{w}{2}\right),\;=\frac{\left|h-w\right|}{w+h}\times 100\%\left(h\neq \frac{w}{2}\right).$$

These equations estimated that the optimal *w* was 133 ≤ *w* ≤ 166 μm for the present study. The process window was derived for the process conditions belonging to this appropriate range of *w*. To evaluate the process window, cubic samples of 5 × 5 × 5 mm^3^ were printed using specified process conditions, and the product quality was evaluated. To analyze the defect rate of the cubic sample, imageJ (Version 1.51j8v, Wayne Rasband, Bethesda, MD, USA) was used to analyze six parts of the cross-section specimen under an optical microscope at a magnification of ×150. Defects in the cubic samples were analyzed using a scanning electron microscope (JSM-7200F, JEOL Ltd., Tokyo, Japan) and Energy-Dispersive Spectroscopy (EDS, NanoAnalysis, Oxford instrument, Oxford, UK).

## 3. Results and Discussion

### 3.1. Characteristics of the Single Scan Tracks

**Figure 2:**
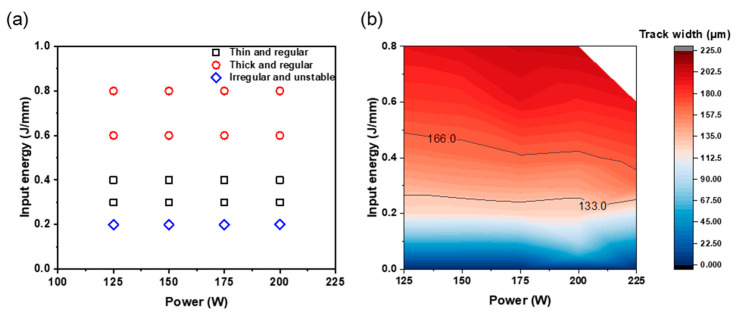
(**a**) The morphology of single scan track according to laser power and input energy at 100 μm beam size. (**b**) Contours map of track width at 100 μm beam size.

The surface morphology of the SST was investigated to understand how the process conditions affect it. The surface morphology of the SST was shown as a process window map according to the process conditions (Figure 2a). The SST surface morphology can be classified into irregular and unstable, thin and regular, and thick and regular according to *E*. ‘Thin’ was defined as 133 ≤ *w* ≤ 166 μm, and ‘thick’ was defined as >166 μm [12]. ‘Regular’ describes passages in which the track width was constant; ‘irregular’ describes the others. The value of *w* of the SST varied accordingly with *P* and *E* (Figure 2b). *P* did not significantly affect the *E* required to form *w* = 133 μm, which corresponds to a 25% overlap. An increase in *P* caused a decrease in the *E* required to achieve *w* = 166 μm, which corresponds to a 35% overlap. The optimal *E* to form 133 ≤ *w* ≤ 166 μm was about 0.24 J/mm to 0.49 J/mm in the process optimization map at *Bs* = 100 μm.

**Figure 3:**
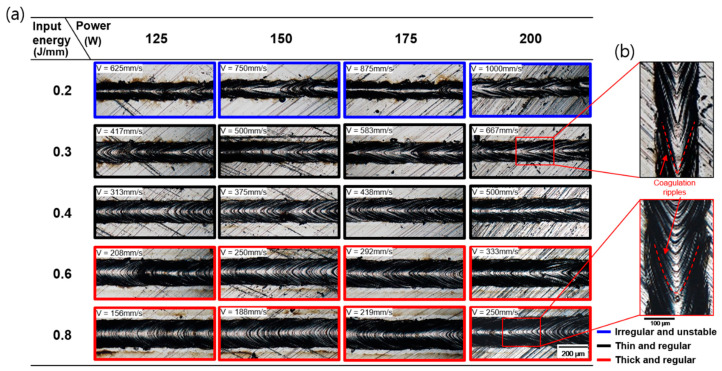
(**a**) Surface morphologies of single scan tracks according to laser power and input energy at beam size 100 μm. (**b**) The shape of coagulation ripples on the surface at different scan speed.

The top surface image of the SST was formed according to *P* and *E* conditions at *Bs* = 100 μm (Figure 3). The surface images of SSTs were classified according to their type of morphologies. There was no change in the surface morphology according to the change in laser power at the same input energy. The irregular and unstable surface morphology of were commonly observed at *E* = 0.2 J/mm. The formation of a continuous track means that applied *E* was sufficient to completely melt the powder. However, tracks formed at *E* = 0.2 J/mm developed irregularities, distortion, and necking. Distorted and necked tracks reduce the final product’s surface quality and density. A 3D image analysis was performed using a digital optical microscope to analyze the surface shape and profiling data of the SSTs.

**Figure 4:**
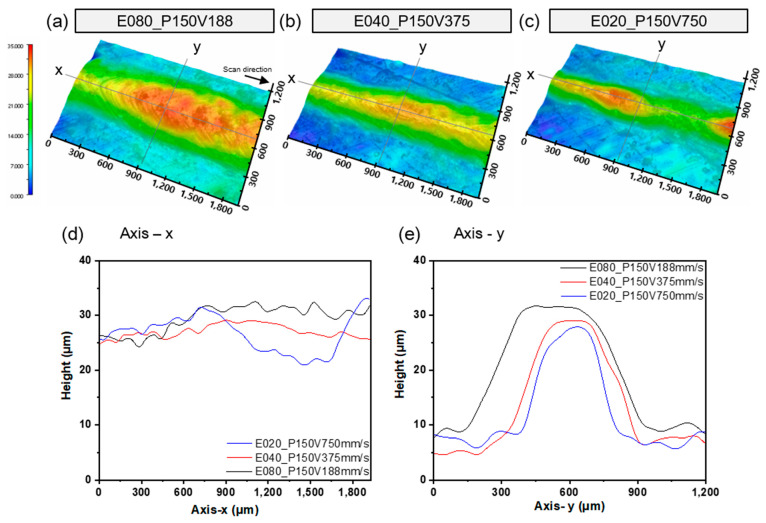
Surface topography of single scan tracks with different input energy, (**a**) 0.80 J/mm, (**b**) 0.40 J/mm, (**c**) 0.20 J/mm, at laser power 150 W. Profiling along (**d**) the laser scan direction (*x*-axis) at center of track, and (**e**) width of track (*y*-axis).

The surface topography in Figure 4 was measured for SSTs that had been formed at *V* = 188, 375, or 750 mm/s with *P* = 150 W. Average heights and standard deviation at an SST of length 1.8 mm on the x-axis were 29.5 ± 2.4 μm at *V* = 188 mm/s, 27.1 ± 1.2 μm at *V* = 375 mm/s, and 26.6 ± 3.3 μm at 750 mm/s (Figure 4d). The height of the deposited tracks has a significant impact on the quality of bonding of neighboring layers. If the track height deviation is large, it means that the melting of the powder is insufficient. This can easily form defects. Therefore, the height of the melt pool needs to have an appropriate height and low deviation.

Surface roughness tends to increase as the exposure time decreases due to an increase in *V*. The scan speed markedly affects the characteristics of the molten pool [21]. When a continuous SST is produced but appears irregular and unstable, reducing the *V* while maintaining the same *P* can lead to the formation of a more regular melt pool. However, even when a continuous track is formed, it can still exhibit irregularities. This is attributed to the surface tension, which induces lateral flow at the edges of the melt pool, functioning to minimize the surface tension [21]. To prevent unstable melt pool from inadequate reflux, sufficient *E* is required in the melt pool by reducing the scan speed or increasing the power (Figure 3). The balling and instability of the melt pool can be seen as being the result of Rayleigh–Plateau capillary instability [22]. A powder bed can be modelled as a liquid metal cylinder with diameter *D* and length *L* [1]. The condition for instability of such a cylinder is satisfied when its harmonic disturbances have wavelengths comparable to *L* or when *L*/*(Dπ)* > 1. The process conditions such as *E*, *P*, and *V* in L-PBF affect melt pool shape. For example, higher scan speeds increase the melt pool length and decrease its width. The shape of coagulation ripples on the surface at different scan speed are shown in Figure 3b. The ripples pattern depends on process parameters. As the *E* increases with the decreasing scan speed, the structure changes from a narrow and long slender shape to a short and nearly spherical shape. Thus, the instability of the melt pool can be reduced by decreasing the scan speed due to the reducing *L* and increasing *w* of the melt pool, as shown in Figure 4.

At *E* = 0.3 and 0.4 J/mm, the tracks were thin and regular. This result suggests that Fe-Si powder melted efficiently. An increase in *w* causes an increase in the overlap rate, and therefore an increase in local energy, which can promote the evaporation of the molten metal to yield pores in the molten pool. Conversely, a decrease in *w* causes a small overlap rate and a decrease in local energy; this change may cause the formation of less-molten regions between adjacent tracks, thereby decreasing the density and yield strength of the product.

At *E* > 0.6 J/mm, thick and regular track shapes appeared, as shown in Figure 3. The overall thickness of the formed track tended to increase as the input energy increased in Figure 4e. At *E* = 0.2 J/mm, the track was irregular and narrow with a large height deviation. At *E* = 0.4 J/mm, the track was the most regular. At *E* = 0.8 J/mm, the track is higher than other tracks, and the surface of the track side is higher than the center of the track. A higher *E* with increasing *P* or decreasing *V* causes the peak temperature to exceed the boiling point of the metal. A high *E* generates excess heat and increases the temperature gradient within melt pool, which strengthens the Marangoni effect and the recoil pressure that increases exponentially as the surface temperature increases [4]. The occurrence of a double-crest surface can be described with the recoil pressure [23]. The high *E* due to the high *P* and low *V* leads to large recoil pressure gradient. So, a double-crested surface can occur. The appropriate recoil pressure pushes the melt pool to spread well. However, if the recoil pressure gradient is too high, the center of the melt pool can be pushed toward the edge of the melt pool and accelerate the penetration of the melt pool, causing a keyhole. This may cause a keyhole pore, and this phenomenon can reduce the composition of the material in the molten pool and blow away the powder around the pool so that insufficient powder remains for adjacent tracks [6]. Many investigators have suggested that the applicant of a very high *E* results in an enormous melt volume [24,25]. This can lead a significant reduction in the flatness of the surface of melt pool, resulting in numerous pores in the fabricated part.

### 3.2. Effects of Process Parameters on Track Widths of SSTs

**Figure 5:**
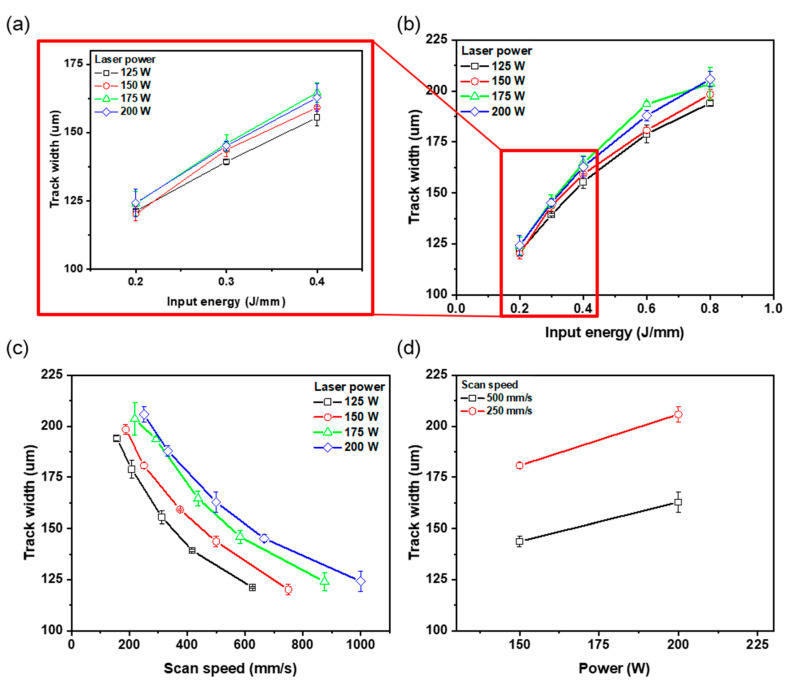
Effect of (**b**) input energy, (**c**) scan speed, and (**d**) laser power of track width. (**a**) Enlargement of region of input energy 0.2~0.4 J/mm.

The value of *E* is determined by *P* and *V*, so both parameters are affected *w* (Figure 5a,b). The value of *w* tended to increase as *E* increased when 125 ≤ *P* ≤ 200 W. This result is consistent with previous reports [17,26,27]. At *E* = 0.2 J/mm (Figure 5a,b), variations in *P* had little effect on *w*; the maximum difference was 4.1 μm. At *E* = 0.8 J/mm, variations in *P* had affected *w* strongly; the maximum difference was 11.9 μm. The value of *E* had a direct influence on the shape and size of melt pool and the spreading properties of liquid metals [26]. Because an increase in *E* affects the increase in the peak temperature of the molten pool, the effects of *P* and *V* on *E* must be distinguished.

At the same *V*, *w* tended to increase as *P* increased (Figure 5c,d). At *V* = 250 mm/s, *w* increased by 25.1 μm from 180.7 μm at *P* = 150 W to 205.9 μm at *P* = 200 W and at *V* = 500 mm/s, while *w* increased by 19.1 μm from 143.7 μm at *P* = 150 W to 162.8 μm at *P* = 200 W. Laser power directly affects the temperature in the area of the powder bed [28]. Increasing *P* can increase the peak temperature, causing the melt’s spreading properties to be improved. The high peak temperature can cause differences in *w* even for the same dwell time.

As *V* decreased under a constant *P*, *w* of the SST increased. Specifically, at *P* = 125 W, *w* increased by 72.8 μm from 121.2 μm at *V* = 625 mm/s to 194.1 μm at 156 mm/s (Figure 5c). Similarly, at *P* = 200 W, *w* increased by 81.7 μm from 124.2 μm at *V* = 1000 mm/s to 205.9 μm at *V* = 250 mm/s. The change in V alters the temperature distribution by varying the laser exposure time on the molten metal and the area of the powder bed [29,30]. If *V* is excessively high, *E* becomes insufficient, potentially leading to the balling effect and irregular track formation. As discussed in Section 3.1, increasing *V* results in increased melt instability and faster solidification velocity. By reducing the scan speed and extending the dwell time, a continuous and stable track can be formed. A sufficient dwell time is crucial for achieving stable melting and adequate spreading properties. Therefore, under a constant *P*, a decreasing *V* leads to an increase in *w*. This is because the slower scan speed extends the exposure time and enhances the wettability of the molten pool, resulting in a spreading shape of the molten pool.

**Figure 6:**
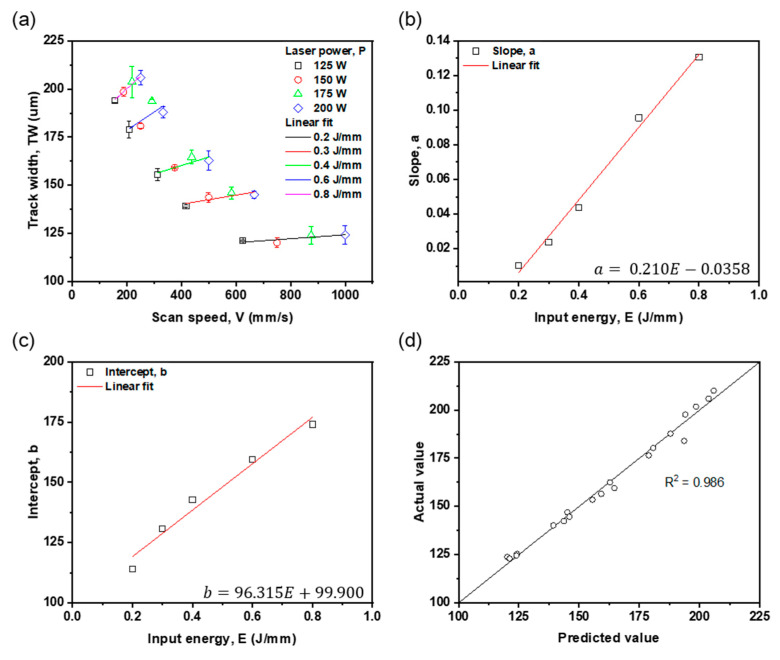
Linear fit of experimentally obtained track width versus (**a**) scan speed and input energy (**b**) slope, (**c**) intercept. (**d**) Comparison between experimental and predicted results.

The relationship between w and process conditions was analyzed using a 2D linear function from the w results obtained experimentally. A linear fit was applied to the *w* data (Figure 6) in which the value of *w* according to *V* was classified according to *E* as

(2) $$\left\{\begin{array}{c} w=aV+b,\\ \;a=0.210E-0.0358\\ \;b=96.315E+99.9.\; \end{array}\right.,$$

The equation *w* = *aV* + *b* was obtained by applying linear fitting to each *w* value formed at 125 ≤ *P* ≤ 200 W at the same *E*. Then, a=0.210E−0.0358 and b=96.315E+99.9 were obtained by performing a linear fit with the slope of the fitting line obtained from each *E* and the y-intercept. The prediction model $w=\left(0.210E-0.0358\right)V+\left(93.315E+99.9\right)$ had a high R^2^ = 0.986. The equation is consistent with the findings of a previous study [31], which used machine learning to find that *w* is most highly correlated with *E*, *V*, and *P* in that order. To further simplify and highlight the relationship between *P* and *V*, we derived another equation: $w=0.210P-0.0358V+93.315\frac{P}{V}+99.9$. The process conditions of L-PBF and morphological data such as *w* and the depth of the molten pool can be applied to construct a process map by applying a constant linear relationship [32].

The prediction model for *w* was verified to have high prediction performance as a function of *V* and *E*. Here, *E* represents the amount of energy per unit area, and *V* signifies the dwell time of heat energy. When *V* is high, a greater amount of energy is required for melting the area of the powder bed. As *E* increases, the depth of the molten pool increases after an initial increase in *w* [33]. This indicates that an increase in *E* enlarges the volume of the melt pool. *V* is closely related to heat transfer and the rate of solidification. An increase in *V* leads to an elongation of the melt pool in the scanning direction, which restricts width growth due to insufficient transverse heat transfer. This phenomenon may result in increased instability of the melt pool. As discussed in Section 3.1, the formation of coagulation ripples on the surface occurs under conditions of a fast *V*. Conversely, when *V* is reduced, heat transfer is facilitated not only in the scanning direction but also transversely, potentially increasing melting in the width direction [33]. *P* also plays a role in determining the *w* of the melt pool. However, increasing *P* tends to deepen the melt pool rather than widen it [34]. Therefore, the prediction model for *w* is highly accurate as it is based on a function of *V* and *E*.

### 3.3. Effects of Process Condition on Cubic Sample Defects

**Figure 7:**
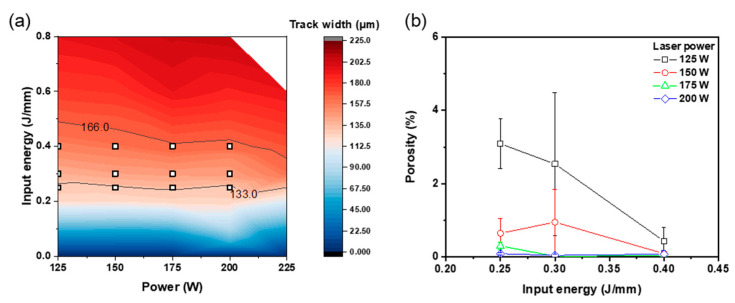
(**a**) Process conditions (squares) applied to cubic samples. (**b**) Effect of laser power and input energy on porosity of Fe-3.4%Si cubic samples.

**Figure 8:**
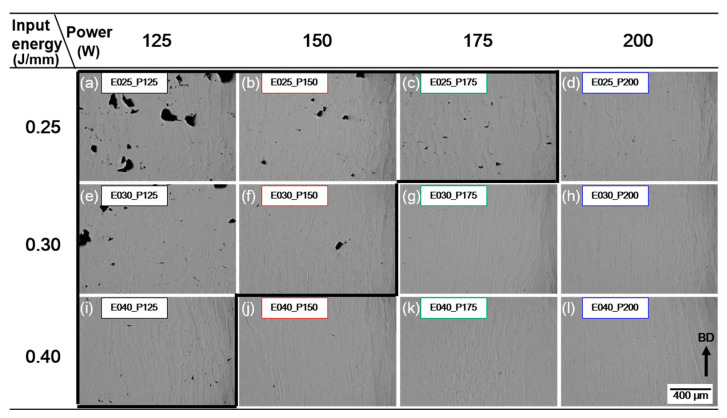
Optical micrographs images of as-printed cubes displaying the porosity according to laser power and input energy. (**a**) *P* = 125 W, *E* = 0.25 J/mm; (**b**) *P* = 150 W, *E* = 0.25 J/mm; (**c**) *P* = 175 W, *E* = 0.25 J/mm; (**d**) *P* = 200 W, *E* = 0.25 J/mm; (**e**) *P* = 125 W, *E* = 0.30 J/mm; (**f**) *P* = 150 W, *E* = 0.30 J/mm; (**g**) *P* = 175 W, *E* = 0.30 J/mm; (**h**) *P* = 200 W, *E* = 0.30 J/mm; (**i**) *P* = 125 W, *E* = 0.40 J/mm; (**j**) *P* = 150 W, *E* = 0.40 J/mm; (**k**) *P* = 175 W, *E* = 0.40 J/mm; (**l**) *P* = 200 W, *E* = 0.40 J/mm.

The cubic samples were manufactured with the proper process conditions based on the track width *w* and surface characteristics of SST for producing high-density samples. The cross-section porosity and defects were assessed by comparing them according to *E* and *P*. The conditions were selected in the process window map derived by the analysis of *w* (Figure 7a), and pores and defects were analyzed (Figure 7b). The porosity of the cubic sample tended to decrease as *E* increased and decrease as *P* increased. An increase in either *E* or *P* led to an enlargement of the width *w* and the overlap rate. This means that increasing *P* or *E* expands the remelting area and mitigates pore formation. Optical micrographs of the polished cubic cross sections (Figure 8) present the effect of the *E* and *P*. At *E* = 0.4 J/mm, the porosity was low regardless of the power. *E* = 0.25 J/mm yielded a low porosity of 0.3 to 0.09% at *P* ≥ 175 W. An analysis of cross-sections of cubic samples showed few crack defects; this result concurs with a previous study [16], which showed that pore defects were formed as major defects. Irregular pores due to the lack of penetration were mainly observed; this type of defect forms due to insufficient input energy (Figure 8a–c,e,f,i). The occurrence of irregular pores decreased as *E* or *P* increased (Figure 8d,g,j). As a result, increasing *P* and *E* as *V* decreases promotes the spread of the melt pool and reduces the formation of pores due to the lack of penetration.

**Figure 9:**
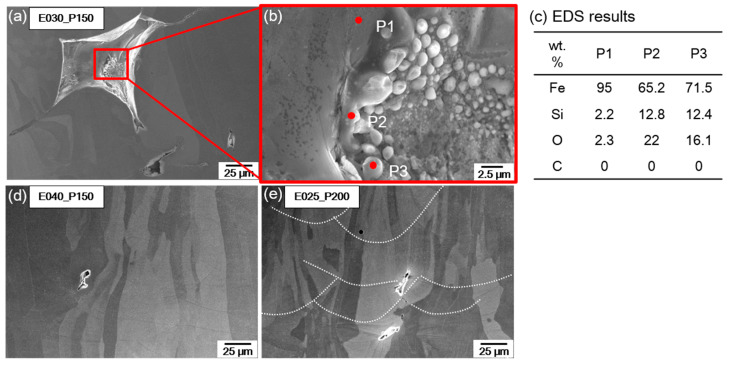
SEM image of the pores formed under input energy condition. (**a**) *E* = 0.3 J/mm, *P* = 150 W; (**b**) magnified image of the inside of pore; (**c**) EDS results at points indicated in (**b**); (**d**) *E* = 0.4 J/mm, *P* = 150 W; (**e**) *E* = 0.25 J/mm, *P* = 200 W.

The pores in the cubic samples were observed using SEM (Figure 9). Sintered powder forms when some powder does not completely melt; consequently, large and irregular defects form, which cause large pores. An EDS analysis of the sintered powder showed that when the input energy was insufficient (150 W, 0.3 J/mm), Si and O content were as high as 12.8 wt.%Si and 22.0 wt.%O in sample P2, and 12.4 wt.%Si and 16.1 wt.%O in sample P3 (Figure 9a,b). When *E* was increased to 0.4 J/mm by decreasing the scan speed while maintaining *P* = 150 W, or by increasing *P* to 175 W while maintaining *E* = 0.3 J/mm, no large and irregular defects were found (Figure 8g,j). This indicates that irregular defects are formed when un-melted and sintered powder exists between melt pools, which can be compensated by increasing *E* through the expansion of the liquid melt pool [35]. Spherical pores were formed inside the molten pool (Figure 9). Spherical pores form when the material volatilizes due to a high *E*. An increase in *E* can increase this process and increase the formation of spherical pores in the melt pool. Increasing *E* can increase the melting of the powder and reduce the unstable molten pool to improve the density of the product but can also result in the formation of cracks or spherical pores. Therefore, process conditions must be optimized. In this study, appropriate process conditions were derived without the cross-sectional analysis of the melt pool, which was deduced by considering the surface characteristics of SST and the appropriate *w* of SST by overlap rate. The defect rate within the cubic sample was evaluated, ultimately leading to the determination of an optimal L-PBF process parameter range that yields porosity levels of ≤1%.

### 3.4. L-PBF Process Map for Fe-3.4wt.%Si

**Figure 10:**
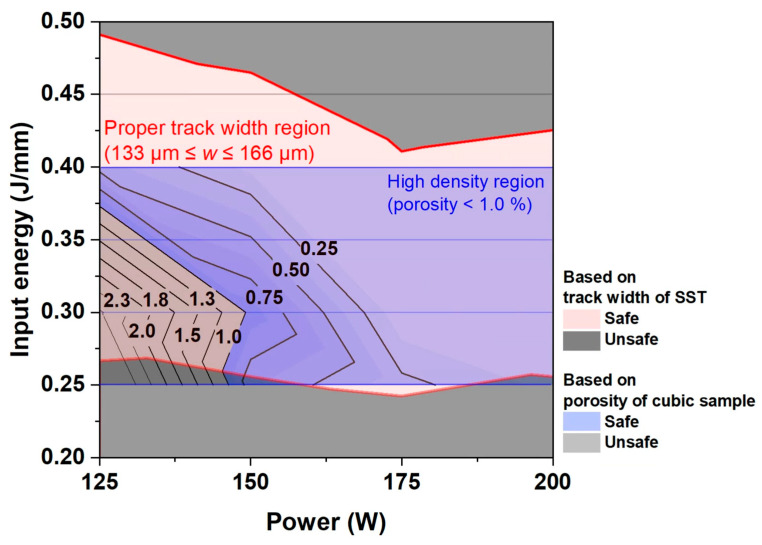
Process parameter map with contours of porosity and red lines showing track width criteria by overlap ratio 25~35%.

The present study introduces an advanced process optimization map (Figure 10), which is based on the overlap rate criterion and porosity in relation to process conditions. This comprehensive map, derived from the analysis of the *w* of SST (Figure 7a) and the porosity of cubic samples (Figure 7b), presents a refined L-PBF process strategy for Fe-3.4Si. The red lines in Figure 10 represent the *w* criterion (133 ≤ *w* ≤ 166 μm), with the optimal process conditions depicted within the red region. In terms of cubic sample porosity, conditions ensuring a high density below the 1% threshold are highlighted in the blue region of Figure 10. While most conditions meeting the *w* criterion also satisfy the porosity criterion, it was observed that conditions with *P* below 150 W and *E* lower than 0.35 J/mm led to more than 1% pore content, indicating a potential for low-quality sample formation. Consequently, a refined process map was developed to ensure the simultaneous achievement of both *w* and porosity criteria. This research not only proposes this refined process map but also accomplishes its formulation through a streamlined analysis approach (Equation (2)), eliminating the need for time-consuming procedures. This strategic framework provides a practical tool for achieving porosity levels ≤1%, enhancing the feasibility of determining optimal L-PBF parameters. This approach optimizes the L-PBF process parameters by identifying conditions necessary for an appropriate SST *w* and ensuring a 25–35% overlap rate of the molten pool. This is based on the fundamental aspects of material behavior under laser processing. Although specific parameters and settings will vary across different materials and processes, our developed strategic approach offers a structured framework for achieving optimal results in various laser-based additive manufacturing applications.

## 4. Conclusions

A study to optimize an additive manufacturing process was conducted by analyzing the w and morphology of the SST generated by laser irradiation on Fe-3.4Si substrate. SST was observed under various combinations of *P* and *V*, which are main factors in the L-PBF process. Then, the results were used to identify the optimal range of process parameters for the L-PBF process of Fe-3.4Si alloy. Cubic samples were produced by L-PBF under these optimal process conditions, and the quality of the samples was analyzed.

(1) At *E* = 0.2 J/mm, obtained using various combinations of *P* and *V*, the molten pools were frequently irregular and unstable. At *E* = 0.3 and 0.4 J/mm, the tracks were thin and regular. At *E* ≥ 0.6 J/mm, the tracks were thick and regular. The SST shapes were greatly affected by *E*, but *P* had relatively small effects. This result suggests that efficient Fe-3.4Si powder dissolution occurred at 0.3 ≤ *E* ≤ 0.4 J/mm, and the appropriate *E* can be approximated just by observing the SST surface shape.
(2) Assuming that a melt pool overlap of 25 to 35% is optimal in additive manufacturing, the optimal *w* was estimated to be between 133 μm and 166 μm for *h* = 80 μm. The *w* of SST was analyzed according to the L-PBF process conditions of *P*, and *E*. A process window to achieve optimal *w* of SST was proposed; for the L-PBF of the Fe-3.4Si alloy, this range is 0.24 ≤ *E* ≤ 0.49 J/mm.
(3) The influence of L-PBF process conditions *E* (J/mm), *P* (W), and *V* (mm/s) on *w* formation was analyzed. As *P* increased, *w* increased at the same *E*. As *V* slowed, *E* increased and *w* also increased. The relationship between w and process conditions was described using an analytical model.
  (3) $$w=\left(0.210E-0.0358\right)V+\left(93.315E+99.9\right)$$
  The value of *w* of SST showed a very high correlation with *E* and *V*. The analytical model will help predict L-PBF process conditions to form the desired *w* of the Fe-3.4Si alloy.
(4) Cubic samples were manufactured to verify the process window of L-PBF for Fe-3.4Si alloy. The process conditions for the cubic samples were selected using the appropriate value of 0.24 ≤ *E* ≤ 0.49 J/mm, which was derived from the optimal value of 133 ≤ *w* ≤ 166 μm. Most manufactured cubic samples had porosity < 1%, except at low value of *P* = 125 W. These results confirmed that the reliability of the process window derived from SST *w* data and the surface characteristics of SST was high, and that the appropriate process conditions could be derived without the analysis of the cross-section analysis of melt pool.

## Figures and Tables

**Table 1:** Chemical composition (wt.%).

Sample	Si	C	P	Fe
Fe-3.4Si	3.38	0	0.005	Bal.

**Table 2:** Ranges of process conditions used in this study.

Process Conditions	Values
Power *P*	125, 150, 175, 200 W
Scan speed *V*	156~1000 mm/s
Input energy *E*	0.2, 0.3, 0.4, 0.6, 0.8 J/mm
Beam size *Bs*	100 µm
Hatch space *h*	80 µm
Layer thickness *t*	40 µm

## Data Availability

The datasets used and/or analyzed during the current study are available from the corresponding author upon reasonable request.
